# Design strategies to improve patient motivation during robot-aided rehabilitation

**DOI:** 10.1186/1743-0003-4-3

**Published:** 2007-02-19

**Authors:** Roberto Colombo, Fabrizio Pisano, Alessandra Mazzone, Carmen Delconte, Silvestro Micera, M Chiara Carrozza, Paolo Dario, Giuseppe Minuco

**Affiliations:** 1 Service of Bioengineering, Salvatore Maugeri Foundation, IRCCS Via Revislate 13, 28010 Veruno (NO), Italy; 2 Division of Neurology, Salvatore Maugeri Foundation, IRCCS Via Revislate 13, 28010 Veruno (NO), Italy; 3 ARTS Lab Scuola Superiore Sant'Anna V.le Piaggio 34, 56025 Pontedera (PI), Italy

## Abstract

**Background:**

Motivation is an important factor in rehabilitation and frequently used as a determinant of rehabilitation outcome. Several factors can influence patient motivation and so improve exercise adherence. This paper presents the design of two robot devices for use in the rehabilitation of upper limb movements, that can motivate patients during the execution of the assigned motor tasks by enhancing the gaming aspects of rehabilitation. In addition, a regular review of the obtained performance can reinforce in patients' minds the importance of exercising and encourage them to continue, so improving their motivation and consequently adherence to the program. In view of this, we also developed an evaluation metric that could characterize the rate of improvement and quantify the changes in the obtained performance.

**Methods:**

Two groups (G1, n = 8 and G2, n = 12) of patients with chronic stroke were enrolled in a 3-week rehabilitation program including standard physical therapy (45 min. daily) plus treatment by means of robot devices (40 min., twice daily) respectively for wrist (G1) and elbow-shoulder movements (G2). Both groups were evaluated by means of standard clinical assessment scales and the new robot measured evaluation metric. Patients' motivation was assessed in 9/12 G2 patients by means of the Intrinsic Motivation Inventory (IMI) questionnaire.

**Results:**

Both groups reduced their motor deficit and showed a significant improvement in clinical scales and the robot measured parameters. The IMI assessed in G2 patients showed high scores for interest, usefulness and importance subscales and low values for tension and pain subscales.

**Conclusion:**

Thanks to the design features of the two robot devices the therapist could easily adapt training to the individual by selecting different difficulty levels of the motor task tailored to each patient's disability. The gaming aspects incorporated in the two rehabilitation robots helped maintain patients' interest high during execution of the assigned tasks by providing feedback on performance. The evaluation metric gave a precise measure of patients' performance and thus provides a tool to help therapists promote patient motivation and hence adherence to the training program.

## Background

Recent epidemiological data point to an increasing trend in prevalence of stroke and this fact has prompted novel treatment approaches based on robot-aided neurorehabilitation. Many researchers using these new rehabilitation tools have investigated upper limb rehabilitation effects by means of detailed kinematic analyses before and after treatment. In particular the MIT-Manus [1-3] and Mirror-Image Motion Enabler (MIME) robots [4,5], which were developed for unrestricted unilateral or bilateral shoulder and elbow movement, show that recovery can be improved through additional therapy aided by robot technology. The ARM guide [6], which assists reaching in a straight-line trajectory, and the Bi-Manu-Track [7], which enables active and passive bilateral forearm and wrist movement, show also that use of simple devices makes possible intensive training of chronic post stroke subjects with positive results in terms of reduction in spasticity, easier hand hygiene, and pain relief. The Gentle/s system [8] is an appealing device that, by coupling models for human arm movement with haptic interfaces and virtual reality technology, can provide robot mediated motor tasks in a three dimensional space. Finally, a robot device based on recent studies of neuro-adaptive control, has been used to generate custom training forces to "trick" subjects into altering their target-directed reaching movements to a prechosen movement as an after-effect of adaptation [9]. This system applies a form of "implicit learning" for teaching motor skills, so demonstrating that it is possible to learn at a quasi-subconscious level with minimal attention and less motivation than more explicit types of practice like pattern tracing.

Motivation is an important factor in rehabilitation and is frequently used as a determinant of rehabilitation outcome [10]. In particular, active engagement towards a treatment/training intervention is usually equated with motivation, and passivity with lack of motivation. Consequently, high adherence to a rehabilitation program is seen as indicative of motivation [11]. In addition to personality and social factors the motivation and adherence of patients to robot-aided treatments can be greatly influenced by the design features of the biomedical robot. In particular the difficulty level of the motor task, the awareness of the performance obtained, and the quantity and quality of feedbacks presented to the patient can influence patient motivation and produce different ways of acting and different performances. Environmental demands play a critical role in the determination of how people execute purposeful actions. Environmental features usually influence the choice of motor strategies. These environmental features are referred to as "regulatory conditions". Often in rehabilitation therapy, patients are asked to perform one or two movement patterns repetitively, the goal being to improve motor performance. Persons with hemiplegia need opportunities to practise skills in situations with varying regulatory conditions so that they can develop motor schemata that are versatile enough to meet the situations they encounter in daily life [12]. Therefore, robot-aided rehabilitation, even if it involves practising only a few articular movements with simple motor tasks, may be considered a tool to help the therapist motivate patients to do voluntary activity with the affected limb when the practice of daily living activities (ADL) is hindered by disability. Robot devices used in neurorehabilitation can offer the patient various different types of feedback and modes of interaction, so influencing the learning process at different levels. It is worth noting that the possibility of assessing patients' performance in a repeatable, objective manner is of great advantage in stroke rehabilitation, and in evaluating treatment effects.

The aim of this paper is to present two rehabilitation robots and the design strategies we implemented in order to boost patient motivation and improve adherence. In addition, we outline a new evaluation metric for quantifying the patient's rate of improvement and allowing a regular review of the performance.

## Methods

### System description

**Figure 1:**
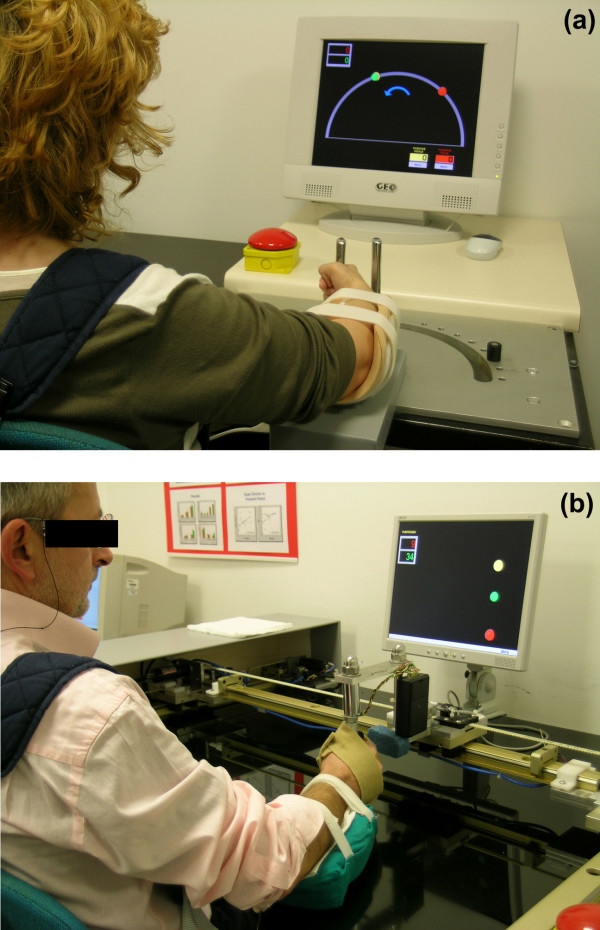
a) One degree of freedom (DoF) robot device for wrist rehabilitation. b) Two DoF robot device for elbow-shoulder rehabilitation.

A one degree of freedom (DoF) wrist manipulator and a 2 DoF elbow-shoulder manipulator were designed for the treatment of our patients (figure 1). Both include an end-effector, normally consisting of a sensorized handle which is grasped by the patient and moved through the workspace of the device (i.e. the horizontal plane). A force/torque transducer is located at the base of the handle near the fixation point so as to provide an estimation of the patient's exerted force/torque in the movement direction. Both devices we developed are admittance control based; this means that the robot detects the force exerted by the patient on the handle and produces a movement in the force direction with a speed proportional to the force amplitude. Three possible control strategies were implemented:

1. completely servo-assisted movements;

2. shared control of the movements (i.e. the system helps the subject to carry out the part of the task he/she is not able to do autonomously);

3. completely voluntary movements.

The devices were applied in the upper limb rehabilitation of two groups of patients with chronic stroke admitted to our Institute for a rehabilitation program. Eight patients (Group 1; aged 66 ± 15 years) were treated using the wrist rehabilitation device and 12 patients (Group 2; aged 55 ± 13 years) with the shoulder-elbow device. A detailed description of the systems can be found in [13,14].

Subjects in both groups were moderate to mildly impaired: inclusion criteria were the presence of a single unilateral cerebrovascular accident and the presence of at least 10° of motion in the treated joints. Mild sensory and visual field impairment and aphasia were not exclusion criteria. Subjects needed to be able to follow the simple instructions of the assigned motor tasks. Patients meeting the inclusion criteria were seen by a professional neurologist who evaluated the patient's neurological status and determined if the patient was medically capable of participating in the study.

The treatment consisted of four cycles of exercise lasting 5 min. each followed by a 3 min. resting period. Subjects were trained twice a day, 5 days a week for three weeks. A practice session preceded the treatment, during which detailed instructions were given to shorten the exercise learning phase. The robot session was fully supervised by the therapist only during the learning phase. Following this, supervision was limited to the patient's connection and disconnection the device and during changes in the difficulty level of the motor task. Patients were seated at the robot desk with their trunk fastened to the back of the chair by a special jacket in order to limit compensation phenomena. A video screen in front of them provided visual feedback in the form of three coloured circles as follows: a) a yellow circle indicated the task's starting position; b) a red circle, the task's target position; c) a green circle, the current position of the handle. The path to follow was a circular arc for the wrist device and a square or a more complex path for the shoulder-elbow device. If, during execution, the patient could not complete the task autonomously, the robot evaluated the current position and, after a resting period of three seconds in the same place, guided the patient's arm to the target position. During the treatment the device provided visual and auditory feedback to the patient to signal the start, the resting phase and the end conditions of the exercise.

### Patient Motivation

Patient cooperation and satisfaction with a training program is essential to achieve successful rehabilitation results [15]. In spite of this, little research has been carried out on motivation in patients with stroke [10]. Several factors can influence patients' motivation and so improve exercise adherence [16]. These include features inherent in the prescribed regimen as well as characteristics related to the patient, physician and therapist [17]. In particular, the major contributors to exercise adherence include simplicity and short duration of treatment [18,19]. Patients who believe that health depends on their own behaviour appear to be more motivated and compliant that those who think that they can do little by themselves to improve their condition and rely on fate, the institution, physician or therapist [20]. Health care providers can usually greatly influence the patient's intrinsic motivation and make exercising more effective [20]. In fact, the patient's perception of therapy, in terms of its relevance to daily needs, the perceived potential to reduce disability and improve quality of life play a role in motivation. Consequently adherence to training is more likely when the therapist gives clear instructions and when the patient understands the rationale and benefits of the prescribed regimen [21].

The introduction of new technologies such as robot devices and virtual reality devices, that partly reduce the patient-therapist interaction, could negatively influence the patient's motivation and hence the crucial questions that arise are: how are these technologies accepted by the patient, and what design and treatment features can positively influence patient motivation? First of all, the initial exercise load should be minimized in order to reduce the start-up effort and decrease the amount of time required for exercise learning. For this reason we developed a special front-end robot interface, thanks to which the therapist could easily select different sequences of targets in the robot workspace so as to propose exercises of a difficulty level tailored to the patient's disability. In addition the front-end interface made it possible to demonstrate the exercise, test the movement range, verify safety of the required movement and adjust robot stiffness. During the learning phase, patients were instructed to make sure they understood how and why the robot-aided exercise needed to be done, and what benefits were expected overall in terms of improvement in daily life activities. No restrictions were placed on the movement in the robot workspace, so that patients could guide the robot handle anywhere their spared function allowed. If the patient could not complete the task the robot assisted in reaching the target.

The robot devices were developed to offer the patient various different types of feedback and modes of interaction, so influencing the learning process at different levels. In fact, in addition to feedback about the position of the handle (green circle), two scores were displayed on the video screen facing the patient during task execution: the first was the score obtained during a single task, the second the score for each 5 min. cycle of exercise. Scores increased only during the patient's voluntary activity, reflecting the proportion of the path travelled by the handle (expressed as a tenth of the total distance between the starting point and the target). They remained unchanged during robot assisted movements. Scores may be very useful in maintaining the patient's motivation high throughout the session, simulating a video-game experience (a higher score indicates a better performance). They are also useful for a quantitative evaluation of the patient's performance. A regular review of performance results also reinforces in patients' minds the importance of exercising and encourages them to continue, so improving their motivation and, hence, adherence to the program. For this reason we developed an evaluation metric that could characterize the rate of improvement and quantify the changes in the obtained performance.

The Intrinsic Motivation Inventory (IMI) is a multidimensional measurement method designed to assess participants' subjective experience related to a target activity in laboratory experiments [22-24]. It consists of a multi-item questionnaire assessing the subject's interest/enjoyment, perceived competence, effort, value/usefulness, felt pressure and tension, and perceived choice while performing a given activity. The interest/enjoyment subscale is considered a self-report measure of intrinsic motivation. The perceived choice and competence concepts are regarded as a positive predictor of intrinsic motivation. The pressure/tension is theorized to be a negative predictor of intrinsic motivation. Past research suggests that order effects of item presentation appear to be negligible. Furthermore, the inclusion or exclusion of specific subscales appears to have no impact on the others [25]. Another important issue of the IMI is that of item redundancy. In fact, items within the same subscale overlap considerably, although randomizing their presentation makes this not relevant to most patients [25]. The full version of the questionnaire includes 45 items and 7 subscales; shorter versions have been used and found to be apparently reliable [26,27]. McAuley et al. assessed the psychometric properties of an 18-item version of the IMI in a competitive sport setting, and found it adequately reliable [26].

In order to evaluate the intrinsic motivation of our patients, we administered a 17-item version to our patients at the end of robot-aided training. Fifteen items assessed the interest/enjoyment, perceived competence, effort/importance, pressure/tension and value/usefulness subscales; each subscale consisted of three items. In addition two items were included to assess if patients experienced pain during treatment with the devices. Each item rated the statement in a range between 1 (not at all true) and 7 (very true). In accordance with the recommendations by the authors of self-determination theory [25], we randomly distributed the IMI items in the questionnaire and formulated them to fit the specific activity of robotic rehabilitation. The items were translated into Italian by a professional translator. To our knowledge, the IMI has never been used to measure motivation in patients after stroke. For this reason we carried out a preliminary principal components factor analysis on a sample of subjects with chronic stroke to explore the validity of the 15-item motivation questionnaire in this patient category. Four independent components resulted from the analysis. As mentioned, two additional items explored the presence/absence of pain. The pain subscale was obtained by averaging the scores of the two items. Thus six dependent variables were obtained from the 17 items. Details about the validation of the IMI questionnaire in patients after stroke will be the object of publication elsewhere.

### Evaluation Metric

No baseline phase was carried out prior to the study with the robot. A standard assessment procedure was used at the start and end of treatment for both groups. This procedure included the upper limb subsection of the Fugl-Meyer scale modified by Lindmak (range: 0–115) [28,29] and the Motor Power Score (range: 0–20) [30,2] that measures strength in proximal muscles of the arm, specifically grading shoulder flexors and abductors and elbow flexors and extensors on a standard 0–5 point scale.

In addition we devised a new evaluation metric based on parameters measured by the robot devices, of use both for motor deficit evaluation and monitoring of patient performance during treatment.

Robot score: the line between the starting point and the target (theoretical path) of a single reaching movement was divided into ten segments (scoring segments). For each point of the actual reaching path, the intersection between the theoretical path and its perpendicular line passing through that point was found. The score increased when (with movement executed by voluntary activity) the point fell in a new scoring segment. If the patient was unable to complete the motor task the robot would guide the patient's limb to the target and the score remained unchanged. When the difficulty level of the task was changed by extending the range of reaching, the 10 scoring segments were altered accordingly. The single task score was obtained by summing the scores obtained in each point to point reaching movement of the task (e.g. four reaching movements in the case of a square). The cycle score was obtained by summing the scores obtained in the tasks executed during each cycle of exercise lasting 5 min. Finally, the Robot score was obtained by averaging the four cycle scores obtained in the training session.

Performance Index: in the case where a patient obtained a maximum score, the motor task was changed extending the range of movement required. The time course of the patient's performance was then obtained simply as the product of the Robot score and difficulty level of the task.

Active movement index: in order to quantify the patient's ability in executing the assigned motor task without robot assistance, we introduced the Active Movement Index (AMI) based on the following formula:

*AMI *= *RS*/TS * 100     (1)

where RS is the Robot score obtained by the patient during the task by active movement, and TS is the theoretical score if the patient completed all tasks by means of voluntary activity.

Mean Velocity: with both devices it was possible to record the current position of the handle. In this way the mean velocity of the handle during the task could be computed. Several papers have shown that the movement during a motor task is the combination of a sequence of sub-movements with a bell-shaped velocity profile [31]. In addition it has been demonstrated that such components are clearly distinct at the beginning of treatment (jerky movements) so resulting in a low mean velocity value, and tend to merge in the course of treatment so producing a smoother movement [1,32]. As a consequence, the mean velocity produced during movement at the end of treatment has a value higher than that at the beginning of treatment. Mean velocity can thus be considered as a measure of smoothness. However two different smoothness 'scenarios' could theoretically have the same mean velocity: i.e. a subject moving slowly without a lot of variation in the speed profile might attain the same mean velocity as one who starts and stops frequently; but the resulting smoothness values should be quite different. For this reason, given the many-faceted aspects represented by the mean velocity, we decided to consider this metric as a distinct component of motor performance evaluation.

This parameter in combination with the session score is very useful for deciding when a change in level of difficulty of the motor task is required. In fact, if during the course of training the patient was able to complete the task with a score close to the maximum (AMI >90%) and a mean velocity close to 50% maximum velocity of the exercise, the therapist increased the difficulty level of the task, extending the path to be covered and/or changing the reaching point sequence.

Movement accuracy: the accuracy of the movement was assessed by the following formula:

MD=(∑i=1n|di|)/n     (2)
 MathType@MTEF@5@5@+=feaafiart1ev1aaatCvAUfKttLearuWrP9MDH5MBPbIqV92AaeXatLxBI9gBaebbnrfifHhDYfgasaacH8akY=wiFfYdH8Gipec8Eeeu0xXdbba9frFj0=OqFfea0dXdd9vqai=hGuQ8kuc9pgc9s8qqaq=dirpe0xb9q8qiLsFr0=vr0=vr0dc8meaabaqaciaacaGaaeqabaqabeGadaaakeaacqWGnbqtcqWGebarcqGH9aqpdaqadaqaamaaqahabaWaaqWaaeaacqWGKbazcqWGPbqAaiaawEa7caGLiWoaaSqaaiabdMgaPjabg2da9iabigdaXaqaaiabd6gaUbqdcqGHris5aaGccaGLOaGaayzkaaGaei4la8IaemOBa4MaaCzcaiaaxMaadaqadaqaaiabikdaYaGaayjkaiaawMcaaaaa@4449@

where MD (Mean Distance) represents the mean absolute value of the distance (di) of each point of the path from the theoretic path. When this parameter approximates zero movement accuracy will be very high.

Normalized path length: the movement's path length was calculated with the following formula:

nPL=(∑i=1n|dPi|)/PLt     (3)
 MathType@MTEF@5@5@+=feaafiart1ev1aaatCvAUfKttLearuWrP9MDH5MBPbIqV92AaeXatLxBI9gBaebbnrfifHhDYfgasaacH8akY=wiFfYdH8Gipec8Eeeu0xXdbba9frFj0=OqFfea0dXdd9vqai=hGuQ8kuc9pgc9s8qqaq=dirpe0xb9q8qiLsFr0=vr0=vr0dc8meaabaqaciaacaGaaeqabaqabeGadaaakeaacqWGUbGBcqWGqbaucqWGmbatcqGH9aqpdaqadaqaamaaqahabaWaaqWaaeaacqWGKbazcqWGqbaucqWGPbqAaiaawEa7caGLiWoaaSqaaiabdMgaPjabg2da9iabigdaXaqaaiabd6gaUbqdcqGHris5aaGccaGLOaGaayzkaaGaei4la8IaemiuaaLaemitaWKaemiDaqNaaCzcaiaaxMaadaqadaqaaiabiodaZaGaayjkaiaawMcaaaaa@4945@

where dPi is the distance between two points of the patient's path and PLt is the theoretical path length, i.e. the distance between the starting point and the target. This parameter is a measure of the efficiency of the movement.

## Results

### Clinical scales results

The robot-assisted therapy was well accepted and tolerated by all patients. Group 1 showed a significant improvement (p < .05) in the Fugl-Meyer scale modified by Lindmark. Because the Motor Power Score evaluated only proximal muscles no changes were found in this group of patients.

Group 2 showed a significant increase in Motor Power score, and in the Fugl-Meyer scale. Details are reported in Table 1.

**Table 1:** Pre and Post treatment values of robot measured variables and clinical scales obtained in patients of Group 1 and Group 2

	Pre	Post	Change	p Value
*Group 1 (n = 8)*				
Robot Score (RS)	110.45 ± 64.54	163.74 ± 74.53	53.29 ± 40.11	0.01
Performance Index (PI)	110.45 ± 64.54	199.73 ± 123.72	89.28 ± 99.53	0.04
Active Movement Index (AMI)	54.13 ± 29.14	72.02 ± 24.43	17.88 ± 22.32	n.s.
				
Motor Power Score (0–20)	13.67 ± 3.31	14.40 ± 2.84	1.13 ± 0.23	n.s
Fugl-Meyer (0–115)	70.43 ± 10.63	75.71 ± 9.34	5.29 ± 4.39	0.04
				
*Group 2 (n = 12)*				
Robot Score (RS)	204.90 ± 90.43	539.70 ± 248.59	334.80 ± 241.98	0.01
Performance Index (PI)	204.90 ± 90.43	1006.3 ± 693.4	801.38 ± 671.98	0.02
Active Movement Index (AMI)	76.57 ± 16.89	95.57 ± 7.28	19.00 ± 16.01	0.01
Mean Velocity (VM)	32.84 ± 10.32	61.55 ± 17.55	28.71 ± 16.92	0.01
Mean Distance(MD)	20.74 ± 10.72	12.42 ± 5.45	-8.32 ± 8.68	0.01
Normalized Path Length (nPL)	1.81 ± 0.59	1.51 ± 0.74	-0.30 ± 0.69	0.01
				
Motor Power Score (0–20)	12.00 ± 2.41	13.40 ± 2.74	1.40 ± 0.77	0.01
Fugl-Meyer (0–115)	61.00 ± 8.17	65.67 ± 10.18	4.66 ± 5.02	0.01

### Evaluation metric results

**Figure 2:**
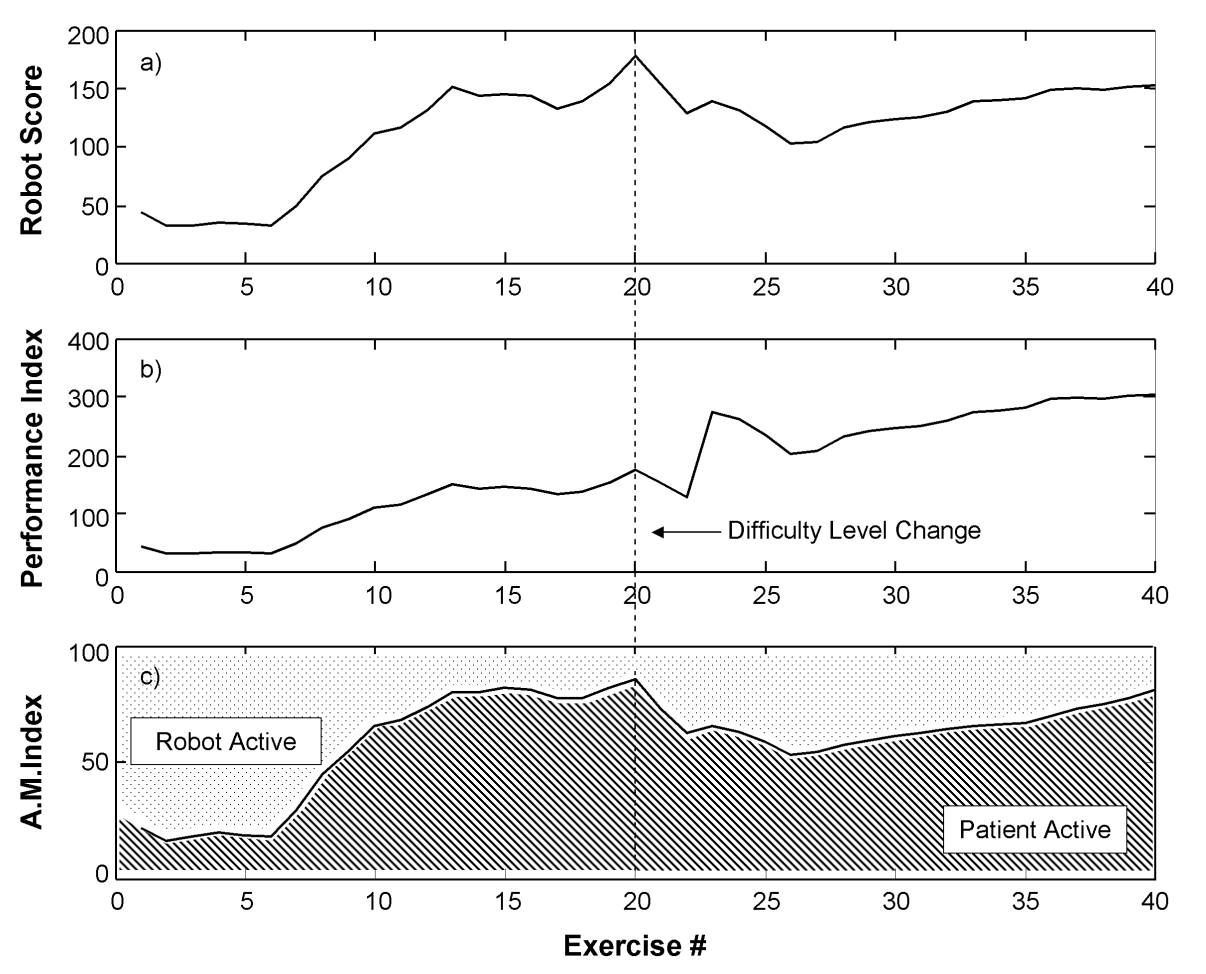
Time course of the robot measured parameters in a representative patient treated by the wrist rehabilitation device.

Figure 2 shows a typical example in one patient of the parameters employed for motor performance evaluation in the application of the wrist robot device.

Panel a) illustrates the Robot score parameter; panel b) illustrates the performance index obtained by multiplying the robot score by the difficulty level of the exercise; panel c) illustrates the active movement index measuring the mean percentage of the patient's voluntary activity exerted during a training session.

The AMI parameter shows that at the beginning of treatment the patient was able to complete only 20% of the motor task without robot assistance. The score subsequently increased to reach a maximum half-way through treatment. At this point the therapist decided to increase the difficulty level of the task. The score temporarily declined because the patient once again needed assistance from the robot device. Then voluntary activity gradually increased again. After 40 training sessions the patient was able to complete 90% of the motor task through voluntary activity. The area under the plot in panel c) represents the patient's activity during training, the area above the plot, the robot's activity.

**Figure 3:**
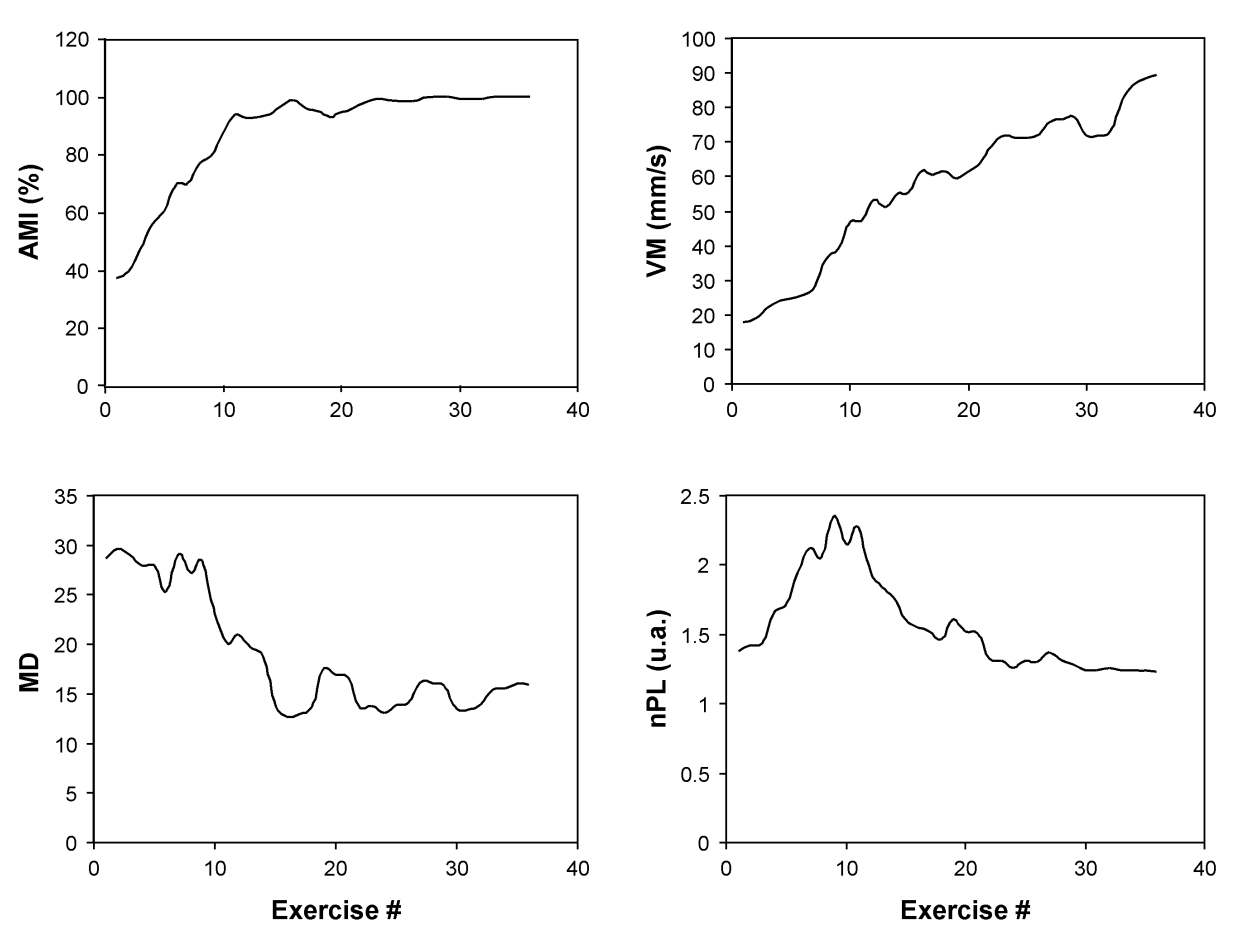
Time course of the robot measured parameters in a representative patient treated by the elbow-shoulder rehabilitation device.

Figure 3 reports an example of the parameters obtained in a chronic post-stroke patient treated with the elbow-shoulder device. It can be seen that the active movement index increased up to half-way through treatment at which point the patient was able to complete the motor task. The mean speed was constantly increasing, indicating a continuous improvement of the patient's performance throughout the treatment. The mean distance and normalized path length decreased, thus showing an improvement in both accuracy and efficiency of movement.

The figures presented cover a wide spectrum of trends encountered with patients involved in this study.

Table 1 summarises the mean values ± standard deviations of PRE and POST treatment clinical variables and robot measured parameters, their changes and the p value of the PRE vs. POST comparison. In Group 1, the robot score and performance index improved significantly. The AMI parameter showed a non significant increase probably due to the small number of subjects. In Group 2, all robot measured parameters and clinical scale values showed a statistically significant change. In particular, the Robot Score, Performance Index, AMI, and Mean Velocity increased after treatment; Mean Distance and Normalized Path Length decreased after treatment, so indicating an improvement in, respectively, accuracy and efficiency of movement.

**Figure 4:**
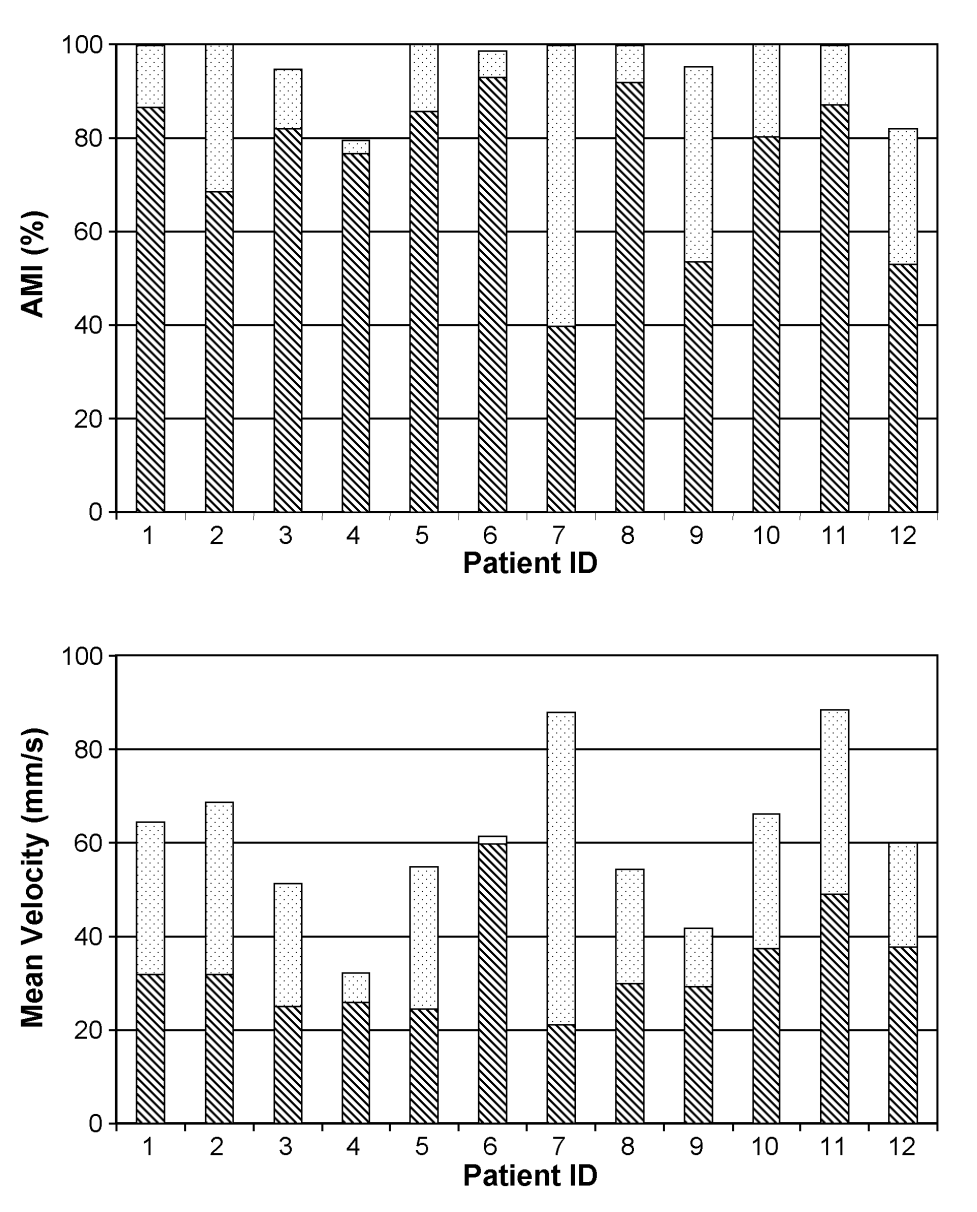
Single subject analysis for the AMI and Mean Velocity parameters. Each bar reports the mean value obtained by the patient at the 3rd training session (hatched area) and the change obtained at the end of treatment (dotted area = significant change, white area = non significant change).

In addition single subject analysis was carried out for the AMI parameter and Mean Velocity of the patients treated with the shoulder-elbow device. Considering that our patients executed many reaching sequences during a training session (on average between 10 and 20 depending on the session number, level of disability, type of task, etc.), we were able to compare data obtained at the third and at the last training session for each subject using Student's t-test for repeated measures. This allowed a single subject evaluation of the change obtained in the measured parameters. Figure 4 reports the mean values obtained by each patient at the third training session (hatched area) and the change at the end of treatment (dotted area = significant change, white area = non significant change). After robot treatment all Group 2 patients showed a significant increase in the AMI and all patients but one (#6) a significant increase in mean velocity.

These results confirm the improvement of performance obtained by our chronic stroke patients after robot-aided rehabilitation.

### Intrinsic Motivation Inventory results

Due to the fact that it had been just recently introduced to our institution, the IMI questionnaire was administered only to a subgroup of Group 2 patients; therefore this study should be considered as preliminary to a more extensive clinical study.

Table 2 reports the mean values and standard deviations of five pre-selected subscales of the IMI questionnaire and pain subscale, evaluated in 9 of the 12 patients treated with the elbow-shoulder rehabilitation device. The interest/enjoyment subscale, i.e. a self-report measure of intrinsic motivation, obtained a high score and a low standard deviation. This suggest that our patients found the robot therapy very interesting.

**Table 2:** Subscale findings of the Intrinsic Motivation Inventory questionnaire evaluated in patients treated with the elbow-shoulder rehabilitation device (subscale range = 1 – 7)

*Group 2 (n = 9 out of 12)*	*Score (Mean ± S.D.)*
Interest/Enjoyment	6.00 ± 1.49
Perceived Competence	4.59 ± 1.89
Effort/Importance	6.70 ± 0.72
Value/Usefulness	6.15 ± 1.38
Pressure/Tension	2.26 ± 2.07
Pain	2.39 ± 2.28

The perceived competence subscale resulted in a mid score (subscale value = 4.6). This result is not surprising because of the different levels of disability of our patients. In fact, less compromised patients should obtain a better performance, and therefore consider themselves more competent in executing the exercise than more compromised patients.

Also the effort/importance and value/usefulness subscales obtained a high score and very low standard deviation so indicating that patients were highly motivated in the execution of this type of treatment, and were satisfied with the results obtained. In particular they perceived that the learning phenomenon obtained by repeating a movement could produce positive results in improving their disability. The pressure/tension and pain subscales obtained a low score with high standard deviation. This means that the majority of patients did not experience tension or pain during training with the robot device.

Only one patient felt tense during the execution of exercises (she was also under treatment for depression). Two patients showed discrepancy in the response to the pain items. This made us suspect that the formulation of the negative sentence may have been a little confusing so producing an unreliable response.

Table 3 presents the correlation analysis between the parameters included in the evaluation metric and the motivation subscales of the IMI questionnaire. Most of the robot measured parameters included in the correlation analysis showed a weak or no correlation with the interest/enjoyment, perceived competence, effort/importance, value/usefulness and pressure/tension subscales. This result is in agreement with other reports in the literature showing a quite modest correlation between self reported motivation variables and behavioural indices [33]. One might expect that an increase of performance corresponding to an increase of the relative parameter should be reflected by an improvement of motivation and hence show a positive correlation. Conversely a negative indicator of motivation, such as pressure/tension subscale, should be negatively correlated with increasing parameters. Equivalent reasoning but with an inverse correlation should be valid where a decrease of parameter corresponded to improvement of performance. These considerations are verified in table 3 only for correlation values greater than or equal to 0.4, i.e. for moderate correlation between variables [34]. Mean velocity was the only parameter showing a moderate correlation both with the effort/importance and pressure/tension subscales.

**Table 3:** Correlation between parameters evaluating patient's performance and motivation subscales

	Interest/Enjoyment	Perceived Competence	Effort/Importance	Value/Usefulness	Pressure/Tension
RS (+)	-0.088	-0.004	0.315	0.109	-0.309
PI (+)	-0.295	0.277	0.278	-0.071	-0.430
AMI (+)	0.072	-0.215	0.242	0.285	-0.462
VM (+)	-0.193	-0.245	0.482	0.086	-.0527
MD (-)	-0.344	0.201	0.155	-0.399	0.150
nPL (-)	0.084	0.384	0.173	0.098	-0.164

The sign next to each parameter indicates that improvement in performance is reflected by an increase (+) or decrease (-) in the parameter.

## Discussion

The two robots presented fulfill the requirements of our occupational therapists who need, when administering robot-aided therapies, to know which motor tasks are most appropriate for each patient and what difficulty level of the task is suitable for the patient's residual capacity. The user interface of the devices we developed allows easy configuration and adaptation of the tasks. In addition the feedback scores provided to the patient – simulating a video-game experience – may be very useful for maintaining the patient's interest high throughout the training session, improving motivation and resulting in a better performance.

Patient motivation can be modified by a number of processes, such as increasing problem awareness and information in patients, involving them in the design and implementation of the treatment program, enhancing their level of internal control and raising their hope of recovery. Motivation programs are designed with specific interventions targeted to modify these factors. We think that our robot devices and the evaluation metric presented here can provide a further up-to-date tool to help therapists promote patient motivation. Of course the visual feedback interface we adopted is very simple; nevertheless the results of the interest/enjoyment scale for the exercises proposed are reassuring. And it should be stated that the easier the gaming interface, the better understood it is by the patient [35]. On the other hand motivation usually is not a constant factor but a dynamic process; thus the willingness of a patient to adhere to a prescribed treatment may change over time in relationship to many factors, in particular, the efficacy of the rehabilitation strategies adopted.

Training with robot devices constitutes a different form of exposure to enriched environments in that the motor tasks used are specific rather than general. Several reports in the literature have shown that robot devices may contribute to improving and accelerating the various stages of recovery [1-7,36]. In particular the learning process obtained by movement repetition is not a unitary phenomenon but can affect many different components of sensory and motor processing. In normal subjects, the repetition of a task usually improves motor performance in terms of accuracy and speed of movement. In neurological rehabilitation the assessment of motor recovery should also include the smoothness, efficacy and efficiency of the movement. Thanks to the quantitative evaluation metric we developed, the process of post-stroke motor recovery may be precisely characterized and quantified in terms of rate of improvement of the patient's voluntary activity. Moreover, on the basis of the motor learning model, we can speculate that the mechanisms underlying this recovery process and resulting in a voluntary activity increase are likely related to robot induced improvement in accuracy, velocity, strength and range of motion of the paretic upper limb. The evaluation metric presented here makes it possible to precisely plan and, where necessary, modify the rehabilitation strategies so as to improve patient adherence to the assigned motor task and, as a consequence, improve the motor outcome.

Finally, the adherence of our patients to the exercise program using robot-aided neurorehabilitation could not be directly measured in this study. In fact, all subjects included in the study were hospitalized for the robot treatment period; thus, quantification of missed sessions or treatment duration, usually considered a measure of adherence to prescribed home exercise, was not relevant here. In fact, all patients received the same prescribed regimen until discharge and the duration of each training session was established by the device. The fact that robot therapy was well accepted and tolerated by all patients, that the robot-measured parameters showed a statistically significant change, and that the intrinsic motivation scales showed high scores leads us nevertheless to presume that also patient's adherence was very high (confirming this is the fact that there were no drop-outs). In future studies, a fixed session duration could be suggested by the therapist at the start of training, but leaving it up to the patient to decide when to stop therapy. The difference between suggested and actual durations of each treatment session could then be considered as a measure of adherence.

A limitation of this study is that no control group was included. Its inclusion would have allowed comparison of the robot therapies and subsequent subject motivation levels with other interventions, thus identifying any intrinsic motivator as a function of a different extrinsic motivator.

## Conclusion

The design features of the two rehabilitation robots presented here permitted the therapist to easily adapt training to each subject by selecting motor tasks tailored to his/her disability. The scoring of performance incorporated in the two rehabilitation robots, and provided to the patient by visual feedback, allowed us to maintain patients' interest high during the training. Furthermore, the evaluation metric proposed allows a precise measure of the patient's performance so providing the therapist with a tool for implementing reinforcement techniques (such as giving positive feedback and commending patients for their efforts) that can promote patient motivation and enhance adherence to the training program.

## Authors' contributions

RC conceived the study, participated in its design and drafted the manuscript. FP participated in the design of the study, in patient selection and evaluation, and helped to draft the manuscript. CD and AM made substantial contributions to acquisition, analysis and interpretation of data. SM, MCC and PD participated in the design of the robot devices and in the revision of the draft. GM participated in the design and coordination of the study and helped to draft the manuscript.
